# Evolving applications of the egg: chorioallantoic membrane assay and *ex vivo* organotypic culture of materials for bone tissue engineering

**DOI:** 10.1177/2041731420942734

**Published:** 2020-10-20

**Authors:** Karen M Marshall, Janos M Kanczler, Richard OC Oreffo

**Affiliations:** Bone and Joint Research Group, Centre for Human Development, Stem Cells and Regeneration, Institute of Developmental Sciences, University of Southampton, Southampton, UK

**Keywords:** Bone tissue engineering, chorioallantoic membrane, CAM assay, *in vivo*, angiogenesis

## Abstract

The chick chorioallantoic membrane model has been around for over a century, applied in angiogenic, oncology, dental and xenograft research. Despite its often perceived archaic, redolent history, the chorioallantoic membrane assay offers new and exciting opportunities for material and growth factor evaluation in bone tissue engineering. Currently, superior/improved experimental methodology for the chorioallantoic membrane assay are difficult to identify, given an absence of scientific consensus in defining experimental approaches, including timing of inoculation with materials and the analysis of results. In addition, critically, regulatory and welfare issues impact upon experimental designs. Given such disparate points, this review details recent research using the *ex vivo* chorioallantoic membrane assay and the *ex vivo* organotypic culture to advance the field of bone tissue engineering, and highlights potential areas of improvement for their application based on recent developments within our group and the tissue engineering field.

## Introduction

Similar to the placenta in mammals, extra-embryonic structures (allantois, chorion, yolk sac and amnion) are formed within the eggshell of birds.^1^ Fusion of the allantois and chorion, termed the chorioallantoic membrane (CAM), occurs during development and the CAM assay takes advantage of this highly vascular *in vivo* structure by the application of materials, cells or substances to the CAM during growth and development in embryonated eggs.^2^ The CAM is responsible for gas exchange, waste product removal and calcium transport to the developing chick.^3,4^ Commonly, a window is made through the eggshell and inner shell membrane, maintaining the contents in the egg, as per the *in ovo* method, or the egg contents are transferred to a receptacle in the *ex ovo* method, and test materials are placed on the exposed CAM. The eggs are incubated for a period prior to analysing the vascular response of the CAM and chick viability compared with the control eggs, to determine the angiogenic potential, biocompatibility and tissue formed using the test material/cells.

## The CAM

**Figure 1.**
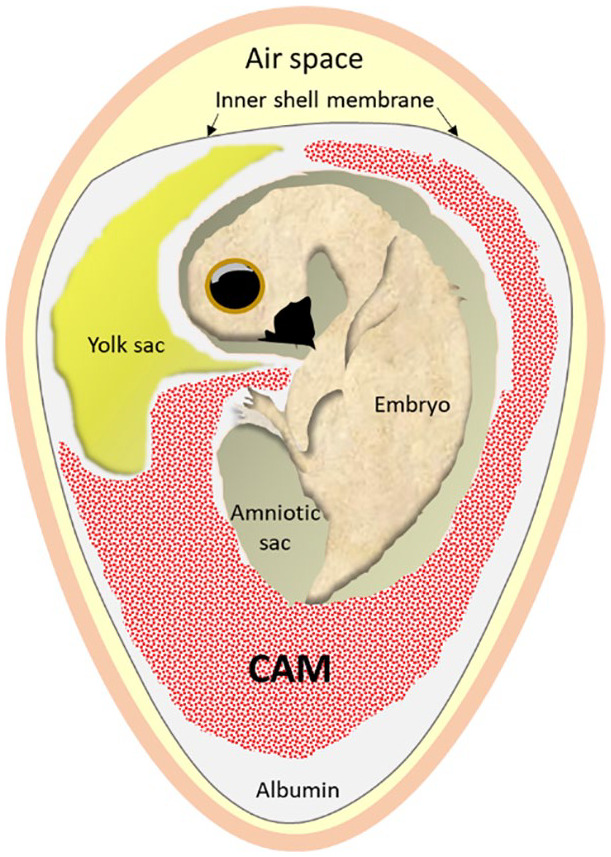
Schematic representation of a chick embryo within the egg. The embryo is attached to the yolk sac, contained within the vitelline membrane, and the allantois at the umbilicus. The allantoic membrane fuses with the chorionic membrane to form the CAM. The outer shell membrane is adhered to the shell, with the inner shell membrane illustrated. The amniotic sac and albumin protect the chick within the egg.

The CAM is the extra-embryonic membrane surrounding the developing chick formed from the fusion of the somatic mesoderm of the chorion and the splanchnic mesoderm of the allantois, beginning on embryonic days (EDs) 4–5 until ED 10, and it is connected to the systemic circulation via two allantoic arteries and one allantoic vein^5–7^ (Figure 1). Histologically the CAM is formed from the ectoderm, mesoderm and endoderm layers, with arteries and veins located in the mesodermal layer when the egg is grown *ex ovo* (outside the shell). However, *in ovo* (within the shell), the allantoic blood vessels form within the allantoic mesoderm and radiate outwards into the chorion ectoderm, towards the shell membrane.^5^ No research could be found detailing the effect this difference in vascular pattern has on the experimental methods used or results obtained. The ability to manipulate the CAM to grow tumours and nourish cells via the systemic and CAM vasculature have made the CAM assay a valuable *in vivo* model. The duration of incubation is sometimes used to describe the developmental stage, however, there are marked differences in chicks’ morphological development, despite identical chronological incubation times, allowing different stages (46 in total) to be described.^8^ Therefore, ED provides a more accurate, simple method of describing the stage of embryogenesis. Importantly, the CAM is considered to lack immunocompetence. Mononuclear phagocytes and reticular cells found in the liver, spleen, yolk sac, bursa, gut and thymus^9^ develop, with T cells appearing at ED 11, while B cells and mononuclear phagocytes appear at ED 12; however, these lymphoid cells are immature, therefore immunocompetence develops at ED 18.^9,10^ This confers a unique advantage – the study and transplantation of xenograft tissues and cells on the CAM, which also can be grown over the longer term by use of multiple CAM experiments in series.^11^ An immune response in the form of graft versus host reactions has been seen when xenogenic leucocytes are intravenously injected, with an inflammatory response and swelling of the chick’s spleen occurring; however, the greatest reaction was to allogenic cells.^12^ Therefore, a reaction can be mounted and perhaps the method of administration also affects the severity of the immune response, for example, intravenous versus topical application. The CAM assay, whether *in ovo* or *ex ovo*, has been found to mount an inflammatory response to materials applied, similar to that seen in mammalian models, allowing extrapolation of results and confirmation of biocompatibility.^13,14^ Bacterial endotoxin, cotton threads and smooth silastic tubing were applied to the CAM with variation in severity of inflammatory response seen with infiltration of cell types such as heterophils (equivalent to mammalian neutrophils), monocytes, macrophages, fibroblasts and giant cells.^13^ The inflammatory response in bone healing is deemed beneficial, linking angiogenesis and osteogenesis; however, it should be short-lived to prevent chronic inflammation and counterproductive tissue damage.^15^ It has been found that material surface characteristics such as porosity, smoothness and shape play a role in cell attachment and the ability for tissue ingrowth, and thus is a factor to consider prior to using the CAM for bone tissue engineering evaluation of materials.^13^ Prior *in vitro* testing of materials, concepts and functions of biomaterials is mandatory and the importance of examining the potential immune response is reviewed by Lock et al.^16^

**Figure 2.**
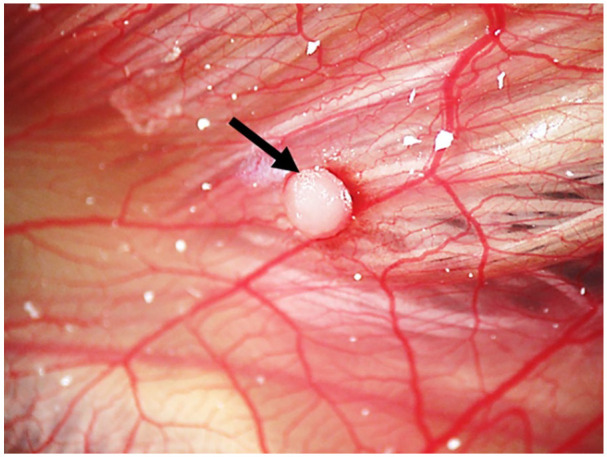
An osteoblast pellet (arrow) on the CAM at ED 18 with the vascular network observed.

The CAM is a highly vascular construct, with vessels forming through a process of sprouting and intussusceptive mechanisms, as the CAM expands rapidly between EDs 9 and 11 and, more slowly, between EDs 12 and 14.^17^ The CAM is fully formed between EDs 8 and 10, with the ability to withstand grafts and scaffolds and in addition, is responsive to stimuli at this time^18^ (Figure 2). Within the CAM are lymphatic vessels, which express vascular endothelial growth factor receptors (VEGFR-2 and VEGFR-3). As would be anticipated, application of vascular endothelial growth factor (VEGF) to the CAM results in angiogenesis, while VEGF-C has been shown to be lymphangiogenic.^19^ VEGFR-2 expression was found to peak at ED 11 and decline towards ED 20, while VEGF expression peaks at EDs 13 and 20, and is lowest at EDs 8 and 15; therefore the timing of application of VEGF to the CAM and consideration of the growth potential should be considered in experimentation.^20^ Hypoxia inducible factor (HIF-1α) expression peaks at EDs 11 and 20 and is lowest at EDs 8 and 15, as HIF-1α induces VEGF transcription.^20^ A cascade involving exogenously applied bone morphogenetic protein (BMP)-4, proto-oncogene c-Src and the VEGFR-2 has been found to promote angiogenesis.^21^

The chick skeleton develops from calcium mobilised predominantly from the eggshell, transported via a calcium binding protein (CBP) in the ectoderm of the CAM, which is closely associated with a Ca^2+^-ATPase, with 85%–95% of calcium previously provided by the yolk sac during the first 10 days.^22–27^ The calcium from the shell is mobilised via the enzyme carbonic anhydrase, at EDs 11 to 12, until hatching occurs at ED 21.^25,26,28,29^ It has been shown that the equator of the egg becomes thinnest as the CAM contacts the shell membranes at this region at EDs 9 to 10, but the membrane cells responsible for enzymatic dissolution are formed at EDs 12 to 14.^30,31^

## The value of the CAM in bone tissue engineering research

The use of the CAM in research was first described in 1911 by Rous and Murphy^32^ for the implantation and study of avian tumours, with further study in 1913 by Murphy^11^ to investigate the biology of cancer cells using xenotransplantation. James B. Murphy showed that Jensen Sarcoma cells could grow on the CAM and the tumour could be transplanted into additional eggs to allow longer term culture for 46 days. In bone tissue engineering, the CAM is used to test growth factors and materials for biocompatibility and the ability of these factors to induce angiogenesis. Angiogenesis is the growth of new blood vessels from existing vasculature.^33^ Such vascular growth is vital when using scaffolds or materials to facilitate the appropriate microenvironment creation and requisite nutrients and oxygen as well as, simultaneously, waste product removal from the healing site by the systemic circulation and lymphatics. Scaffolds required to fill bone defects can be up to several cubic centimetres in size, while the diffusion limit for oxygen is 100–200 µm, and hence the survival of exogenous or host animal/human cells depends on vasculature being formed within the scaffold.^34^ In the normal adult, only 0.01% of endothelial cells undergo division and stimulating the formation of a vascular network can be via the use of growth factors, scaffolds pre-fabricated with endothelial cells or coculture methods prior to implantation, or being able to graft preformed vasculature onto the larger systemic vascular network.^34–37^ Materials which do not support the ingrowth of blood vessels are less likely to integrate with the host bone and show necrosis at the centre, as the initial inflammatory reaction responsible for the angiogenic response creates short-lived blood vessels, with research aimed at creating stable, mature blood vessels *in vivo*.^35,38,39^ Given that the size, stiffness, roughness and porosity of materials and the different types of cells/growth factors incorporated within a scaffold can affect cell attachment, differentiation and survival, the CAM allows these variables to be tested economically and quickly, potentially preventing the use of rodent models in the first instance.^35,40^

## Current common applications of the CAM in research

The main use in research of the CAM is in the assessment of angiogenic,^21,41^ anti-angiogenic or toxic^42^ responses to drugs,^43^ cells, extracellular vesicles^44^ or materials,^3^ biocompatibility of materials,^45^ the growth or treatment of cancer^46–48^ and xenograft application^49–52^ for tissue growth and testing treatment modalities. Oncology research remains, to date, the most common field using the CAM assay.^53^ The CAM assay is also used to test the irritancy and biocompatibility of substances^43^ or materials, which, as a consequence, directly impacts on the welfare of larger laboratory animals, for example, as a substitute for the well-known Draize test in rabbits. The CAM assay is poorly described in veterinary medicine research, although the CAM has been used in feline and canine oncology research,^54^ which could be pivotal in testing treatments for canine osteosarcoma^55^ and translation to human patients and vice versa. Of key interest is the potential of the CAM to provide assessment and visualisation of early biological events, not possible/observed in larger animal models in which experiments are, typically, run over a longer time frame and results gained at a later stage of pathogenesis or healing.^56^ Although usually a short-term assay, longer term mathematical modelling could potentially be developed to predict results, as the CAM has been ‘modelled’ to attempt to understand and simplify cell interactions and the angiogenic response.^57^ Although predominantly thought of as an assay for angiogenesis, the CAM assay can be used to assess bone tissue formation and turnover when bone is applied directly to the CAM.^50,58^ However, growth of bone matrix on a scaffold on the CAM requires *in vitro* application of cells or growth and differentiation of chick cells within the scaffold. *Ex vivo* organotypic culture of chick femurs may be more suitable for assessing tissue formation, due to the large reserve of chick mesenchymal stromal cells able to respond to incorporated cells, growth factors and materials within the bone defect.

### Quantification of angiogenesis

The various experimental methods for stimulating and quantifying angiogenesis have been reviewed by Tahergorabi and Khazaei,^59^ Hasan et al.^60^ and Norrby;^61^ however, the CAM has not superseded these methods in some areas of research, as the *ex vivo* aortic ring assay^62^ and hind limb ischaemia model^63^ are still used, depending on the question being asked and the ultimate research goal. The hindlimb ischaemia model is focused on finding therapies for peripheral vascular disease using methods such as growth factors and stem cells, and would not be a suitable method for scaffold assessment as the model does not imitate the clinical scenario.^59,64^ Methods which involve implantation under the skin or within a body cavity (e.g. dorsal skin chamber, rabbit ear chamber, anterior chamber of the eye, sponge implant assay, disc implantation, Matrigel-plug assay and hollow fibre assay), all involve mammalian test subjects, surgical or invasive procedures, and greater cost and time commitments.^60,61^ These *in vivo* experiments require project and personal licencing from the regulatory body (Home Office in the United Kingdom), use of the PREPARE (Planning Research and Experimental Procedures on Animals: Recommendations for Excellence)^65^ and ARRIVE (Animals in Research: Reporting *In Vivo* Experiments)^66^ guidelines, surgical expertise and have a potentially greater ethical dilemma for researchers regarding their use as primary *in vivo* test subjects. Further to this, bone tissue engineering research usually involves an osteoconductive scaffold to be the underlying frame for which bone will grow onto; therefore, injecting Matrigel subcutaneously does not imitate what researchers are aiming to achieve. However, the Matrigel-plug assay can be used to assess the angiogenic response to growth factors, but again it could be argued that the diffusion of growth factor would not be the same as from the scaffold intended for translation and therefore, the results cannot be extrapolated from this model to future *in vivo* work.^60^ *In vitro* assays assessing parameters such as cell proliferation and tubule formation by endothelial cells or the effects of co-culture and the response to growth factors to form a stable vascular network do not accurately mimic the *in vivo* environment. Hence, after *in vitro* experiments and the observation of encouraging results forming the basis for further investigation, the CAM assay becomes essential in translation of materials for bone tissue engineering prior to rodent subcutaneous models.^67^

## Purpose of this review

The above has touched, briefly, on the biology of the CAM and various fields in which the CAM has an impact and that have enhanced our understanding. In this review, a central focus resides on the regulations and considerations affecting the use of the CAM and *ex vivo* organotypic culture exemplified with recent research in the field of bone tissue engineering. The methodology for establishing the CAM and *ex vivo* organotypic culture of chick femurs and results analysis will be presented and discussed.

## Regulation of CAM use and the application of replacement, refinement and reduction (3Rs)

Use of embryonated chicken eggs for research, in the United Kingdom, is governed by the UK Home Office. The Animals (Scientific Procedures) Act 1986 Amendment Regulations 2012 (SI 2012/3039) amended the Animals (Scientific Procedures) Act 1986 (ASPA) covering European Directive 2010/63/EU. The act details a need for a personal and project licence ‘to carry out regulated procedures on an embryonated bird egg’ if the embryo is manipulated during the first two-thirds of the incubation period and then the embryo is allowed to survive into the final third of the incubation period. In contrast, if the embryo is not allowed to enter the start of the final third of the incubation period, no licence is required.^68^ The incubation period until hatching is 21 days and therefore procedures until ED 14 of incubation are not regulated by ASPA. This time frame makes the CAM assay attractive to researchers, as chick embryos are regarded as lacking pain perception and senescence, leading to reduced concerns over welfare. It is important to note that while procedures on the CAM are often perceived as harmless, *euthanasia* of a live organism is performed, and thus regulations must be followed. Publications can thus, at times, be misleading with reference to no licence being required, but in the absence of clear guidelines around the incubation period or country of origin of research to inform the researcher. Furthermore, the method of euthanasia should be accurately described and humane with the literature published indicating, what could appear, inappropriate methodology – thus, the observed literature statements, taken in isolation, such as ‘cutting their arteries’ would not appear an optimal appropriate description of euthanasia.^69^

The advantages in the application of the CAM for studies are the reduced sentient characteristics and there are reports that electroencephalogram testing indicates a ‘sleep-like state of unconsciousness’ until ED 17.^70^ Interestingly, our own work has shown that when the eggshell window has been opened on the final day of experimentation, on ED 18 for scaffold analysis, a response to light can be observed with repositioning of the chick towards the airspace, ready for hatching, indicating that the chick is fully conscious a few days prior to hatching.^71^ Although the CAM is aneural, the embryo has pain receptors after ED 11 of incubation. Thus, care has to be taken on handling, with euthanasia rapid and in accordance with legislation.^4^ This is corroborated by the nervous system beginning to form at ED 7 with a functional brain present by ED 13.^71^ Therefore, there is understandable concern over any perceived suffering at the time of euthanasia, regardless of whether the experiment falls into a regulated procedure or not.

To date, research that identifies the timing of pain perception in the embryonated chick remains limited. This research understanding is essential for safeguarding the necessary welfare standards when using the CAM assay. To euthanise the embryos, freezing at ED 12 has been reported, but is likely to cause distress for the chick.^72^ Similarly, carbon dioxide gas is unreliable and difficult to ‘dose’; while using a fixative on the CAM is considered painful^71^ and not permitted under ASPA. Others have published alternative approaches – Cirligeriu et al.^56^ describe applying 10% buffered formalin for 30 min to the CAM, on ED 13, which appears counter to EU guidance; however, the method of euthanasia is not described. While anaesthetic overdose or sedation prior to decapitation is advocated, as a humane method of euthanasia,^71^ it is important that such an approach does not engender more distress than the decapitation procedure itself.^68^ Therefore, although a method for pentobarbital injection is described by Aleksandrowicz and Herr,^71^ researchers must be aware that there are regulations concerning the use of barbiturates and administration of sedation or anaesthesia is a regulated procedure, necessitating, typically, a licence or veterinary supervision.

Studies using the CAM assay, or any animals, should, ideally, discuss the ‘3Rs’ led by the National Centre for the Replacement, Refinement and Reduction of Animals in Research. First described by Russell and Burch in 1959 in *The Principles of Humane Experimental Technique*, the definitions for the 3Rs have been scrutinised by Tannenbaum and Bennett^73^ as the original definitions and outcomes are *not defined* the same by many research bodies and therefore are, on occasion, quoted incorrectly in research papers. The 3Rs is based on the concept of distress being inhumane and so conversely, limiting distress would be humane. Russell and Burch, cited by Tannenbaum and Bennet,^73^ state that replacement is the substitution of conscious higher animals for insentient material, and not the complete replacement of animals nor animals of a lower phylogenetic class. Reduction is defined by Russell and Burch as the ‘reduction in the number of animals used to obtain information of a given amount and precision’.^73^ This can constitute using too many animals, with less being satisfactory for a statistically valid result and, conversely, too few animals leading to repetition of the experiment, counter the principle of the 3Rs to reduce distress. Refinement is ‘any decrease in the incidence or severity of inhumane procedures applied to those animals which still have to be used’; however, this is not always achievable depending on the research question.^73^ The CAM assay is especially valuable, given that the CAM confers the ability to reduce the number of conscious, sentient animals in subsequent experiments and therefore limiting pain and suffering. Furthermore, refinement of the technique using the CAM assay may lead to lower numbers of mammals being used, even if the CAM assay is, still, not a total replacement method.^74^ The ability to non-invasively exploit the vasculature of the developing CAM to provide an *ex vivo* biological environment is advantageous compared with invasive surgical methods, for example, the subcutaneous implantation model in rodents, as anaesthesia and surgery are unnecessary in the CAM assay.

## Bone tissue engineering

Bone tissue engineering and the development of real-world solutions to an increasingly prevalent need for bone repair strategies, is of paramount importance due to the economic and quality of life implications of non-healing fractures due to trauma, lifestyle and comorbidities, and in cases of osteoporotic fracture in an increasingly aged population.^75^ Bone tissue engineering often incorporates the ‘diamond concept’, which includes the osteoconductive nature of a scaffold, cells capable of osteogenesis, growth factors for osteoinduction and angiogenesis, and biocompatibility of the material and any breakdown products.^76,77^ The use of materials such as natural or synthetic polymer scaffolds, hydrogels and injectable materials, while using growth factors linked or incorporated into the scaffold by chemical or physical means, attempts to solve the issues around biocompatibility, mechanical resistance and in linking angiogenesis and osteogenesis for successful bone formation. Therefore, a biological, living model which can accommodate such a construct to assess compatibility with cells and allow angiogenesis and tissue formation is ideal, such as the CAM assay. As discussed, *in vitro* results do not always translate into *in vivo* success due to factors such as differences in cell populations present, the immune system, surgical considerations and species differences in efficacy of growth factors used.^78–80^ The interactions between cells and biomaterials which require careful consideration and evaluation have been reviewed by Prezekora,^81^ but it is concluded that *in vitro* results cannot substitute the *in vivo* discoveries. The CAM assay acts as a bridge between *in vitro* and *in vivo* research, screening test materials to save time and expense. The CAM assay is gaining broader use in the assessment of growth factors that are widely used in bone tissue engineering. From a tissue engineering perspective, the CAM contains mesenchymal cells which show ‘stemness’ to differentiate, depending on the environment and factors applied.^82,83^ These cells are important because they allow the interaction between exogenous cells applied to the CAM and resident avian cells, leading to differentiation towards vascular structures, changes in cell phenotype and tumour cell angiogenic mechanisms to be studied.^82,83^ The mesenchymal cells within the CAM can also offer insights into cell differentiation and recruitment when acellular constructs are applied to the CAM surface.^50^

### Growth factor use on the CAM

The angiogenic response using BMP-2, fibroblast growth factor-2 (FGF-2) and VEGF has been measured and a synergistic effect observed using growth factors concurrently *ex vivo*, allowing lower doses to be used than found to be effective *in vitro*.^84^ The application of the CAM to visualise responses compared with other *in vivo* studies, allows application of growth factors sequentially and at differing time points, to enhance the knowledge of the temporal effect of growth factors. Thus, the study by Bai et al.^84^ has implications for the timing and doses of growth factors, which may be efficacious in future rodent and large animal studies.

Growth factor evaluation in bone tissue engineering has examined a number of approaches, including the transfection of cells with the RNA of growth factor proteins to allow sustained release rather than concentrated ‘burst’ or application of the select growth factor. VEGF_165_ RNA was used to transfect MG-63 osteoblast-like cells, which were then used on the CAM. This led to an increased angiogenic response compared with the scaffold (polycaprolactone) alone or recombinant human VEGF_165_ (rhVEGF_165_) applied to the CAM. This formed the foundation of a murine calvarial study to progress the question of bone healing and the angiogenic response. Application of rhVEGF_165_ led to increased bone thickness, while VEGF_165_ RNA showed increased angiogenesis, consistent with findings in the CAM.^85^ This latter study builds on the literature reviewed by Grosso et al.,^86^ discussing the role of VEGF in angiogenesis and osteogenesis.

Further studies investigating the optimal method of presenting growth factors to cells while bound to a scaffold are an expanding area of research. Electrospun polycaprolactone (PCL) scaffolds or ‘nanofibrous substrate’ using anti-BMP-2 and anti-VEGF antibodies to bind BMP-2 and VEGF have been developed by Casanova et al.^87^ Platelet lysate was used to provide endogenous BMP-2 and VEGF at low concentrations, with angiogenesis on the CAM being enhanced when both growth factors were present concurrently.^87^ This method could circumvent issues encountered (and concerns) when using high doses of exogenous growth factors, for example, BMP-2 and reported associated swelling, ectopic bone formation and risk of malignancy.^88^

### Hydrogels on the CAM

A hydrogel formed from Laponite^®^ clay, Gelatin Methacryloyl (GelMA) and 10 µg/mL VEGF was found to have optimal integration and increased blood vessel scoring when compared with each material alone or in combination without VEGF, as the material was capable of sequestering and releasing VEGF on the CAM.^89^ Laponite combined with gellan gum, was found to be a suitable ‘bioink’ in which C2C12 cells were printed successfully within the scaffold.^90^ The scaffold was then tested on the CAM with VEGF (100 ng/mL) and found to be angiogenic when incorporating the growth factor, compared with the control scaffolds.^90^ The CAM assay is ideal for preliminary testing of cell-loaded bioinks due to the lack of immunocompetence. Laponite-alginate-methylcellulose (3-3-3) bioink was applied to the CAM and the addition of VEGF and human umbilical vein endothelial cells (HUVECs) was found to induce a significantly greater angiogenic response than the material alone, without VEGF or without the addition of HUVECs.^91^ BMP-2 (10 µg/mL) was then adsorbed onto ‘3-3-3’ bioink scaffolds for evaluation in a murine subcutaneous implant model and the results showed significantly greater mineralisation in the ‘3-3-3’ scaffolds with or without BMP-2 compared with alginate controls.^91^ Modelling potential pathways of angiogenesis was investigated by Bai et al.^92^ using a polymer scaffold with VEGF and FGF-2 in combination with platelet-derived growth factor (PDGF) in microspheres to create sequential delivery of growth factors. The authors reported a significant increase in mature blood vessels and thickness of the mesodermal tissue on histology. VEGF and FGF-2 delivery can result in unstable ‘leaky’ blood vessels, thus PDGF addition promoted blood vessel maturation. Interestingly, the timing, dose and age of the patient can have an impact on the outcome as VEGF does not appear to have a continuous function in adults, as determined by inactivation in rodent models of varying ages, reviewed by Yancopoulos et al.^93^ Kanczler et al.^94^ determined the effect of VEGF incorporated into a PLA scaffold, with significantly increased blood vessel formation with VEGF compared with PLA scaffold alone and achieved 100% chick survival.

An injectable hydrogel displaying liquid properties at room temperature and a viscous gel at physiological 37°C, with incorporation of stromal cell-derived factor-1 (SDF-1) and VEGF nanoparticles, has been developed by He et al.^95^ to enhance mesenchymal stem cell and endothelial cell migration and angiogenesis. The hydrogel formed from chitosan, sodium β-glycerophosphate and gelatin contained the oppositely charged nanoparticles, to aid release of growth factors and hydrogel stability. The histology of samples from the CAM assay demonstrated enhanced blood vessel ingrowth with the SDF-1 and VEGF nanoparticles within the hydrogel, compared with either growth factor alone or the combined growth factors ‘free’ within the hydrogel.^95^

Malik et al.^96^ employed thyroxine within an injectable, membrane-like hydrogel formed from chitosan, carboxymethyl cellulose and hydroxyapatite to recreate the periodontal ligament. Thyroxine is known to have angiogenic effects via pathways involving angiogenic growth factors.^97^ Hydrogels with 0.1 µg/mL of thyroxine were most angiogenic on the CAM, with blood vessels quantified by blinded observers.^96^

Biodegradable scaffolds composed of reduced graphene oxide nanoparticles within a polyvinyl alcohol (PVA) and carboxymethyl cellulose (CMC) hydrogel were angiogenic at 0.0075% reduced graphene oxide concentration.^98^ Images of the scaffolds on the CAM were taken at ED 8, when implanted and again at ED 10 and the ‘fold change’ in blood vessel number and thickness calculated by Chakraborty et al.^98^ Furthermore, the increase in thickness seen was attributed to arteriogenesis; however, this is the phenomenon of thickening at an arterio–arteriolar junction due to a blockage or change in flow within a vessel^59,99^ and without additional histology, greater interpretation is limited on these findings.

Chitosan and hydroxyapatite hydrogels with varying concentrations of heparin were applied to the CAM;^100^ however, there was a poor survival rate using this *ex ovo* method (described by Mangir et al.^4^) with 40%–50% surviving to implantation of materials and 70%–80% proceeding to survive until sample harvest. With this low and limited survival rate, and low numbers of eggs initially used for each condition, statistical analysis is difficult in this report and the lack of positive controls limits further comparison.^100^

## Application of the CAM to test modified polymer materials

The material of interest alone should be tested for angiogenic properties and biocompatibility with or without growth factor addition. This is attractive, removing the safety risk of adverse effects from growth factors informing future issues with further *in vivo* applications. For example, nanohydroxyapatite rod cores surrounded by a silica sheath incorporated into a gelatin matrix were found to be angiogenic,^101^ as hydroxyapatite is a native component of bone, and silicon has effects on bone formation and remodelling.^102^ Hydroxyapatite-based scaffolds were tested by Tomco et al.^103^ in the CAM and a pig mandibular defect model with the bone marrow stromal cell seeded-scaffold replaced by bone in 9 weeks, although it appeared that the study lacked a ‘no-scaffold’ control to compare normal bone healing rate and morphology in this anatomical region potentially limiting interpretation.

Variations of bioglass materials have been tested on the CAM for angiogenic response, without the use of concurrent cell seeding.^104–106^ ‘Nanobioactive’ glass and alginate with alendronate microspheres incorporating copper or calcium were investigated by Cattalini et al.^104^ The authors observed that the copper containing materials degraded faster and showed higher HUVEC viability. The group then used the copper containing solution soaked into filter paper discs on a quail *ex ovo* CAM assay to evaluate angiogenesis, and found the solution from the bioactive glass/alginate/copper/alendronate material to increase the number of blood vessel ‘branch points’, although this was compared with FGF and alginate ‘extract’ solution alone. Furthermore, the results may have differed if a porous three-dimensional (3D) scaffold had been used on the CAM rather than filter paper discs.^104^

Studies by Augustine et al.^107–109^ focused on the use of metal oxides/hydroxide on electrospun PCL scaffolds to drive angiogenesis, likely through mechanisms involving reactive oxygen species and hypoxia leading to VEGF and other growth factor release from tissues. Zinc oxide (1%wt.)^108^ was observed to be proangiogenic on the CAM and this was further evidenced in a guinea pig subcutaneous implant model. Augustine et al.^109^ stated that since the scaffolds are in contact with the circulation, compatibility using human red blood cells is important to ensure no aggregation or haemolysis occurs, and this was described prior to use in the CAM.^107^ Yttrium oxide was found to be proangiogenic at 1%wt. compared with higher concentrations, and similarly lower amounts of europium hydroxide nanorods incorporated into PCL were optimal for an angiogenic response.^109^ Interestingly, the yttrium oxide or europium hydroxide nanorod-incorporated PCL materials were placed on the CAM for 2 days and imaged on ED 10. Images were taken at 0- and 8-h time points to compare angiogenic response over this short time frame to determine new blood vessel formation.^107,109^

Zinc oxide was used by Rahmani et al.,^110^ incorporated into PCL scaffolds with nanohydroxyapatite to study the osteogenic and angiogenic response with differing ratios of human bone marrow stem cells (HBMSCs) and HUVECs. The scaffolds with or without the incorporation of zinc oxide were then tested on the CAM. The addition of zinc led to increased number of blood vessel branches counted confirming zinc’s angiogenic property.^110^

Synthetic polymer scaffolds with ‘plasma surface modification’ using argon gas, seeded with human adipose stem cells were found to be more angiogenic than when nitrogen or oxygen gas were used, as determined subjectively by immunocytochemistry using VEGF and Laminin markers.^111^

### Effect of the porosity and the characteristics of angiogenic materials on the CAM

Materials derived from natural polymers, with an enhanced textured surface are indicated as more angiogenic^72^ in contrast to smooth, synthetic inert materials.^112^ However, it should be noted that a material which is angiogenic may not give the optimal response if the porosity is sub-optimal.^72^ Thus, studies have examined different-shaped pores. Magnaudeix et al.^113^ reported triangular-shaped pores that aided blood vessel ‘guidance’ and the number of blood vessels growing towards the pores, compared with circular pores. Porous microspheres, which are applicable in an injectable formulation, were found to increase angiogenesis when pre-seeded with embryonic mesenchymal progenitor cells grown in osteogenic media. The surrounding matrix formed by the cells perhaps produced VEGF and held the cells and spheres together as a construct.^114^ ‘Interpenetrating Network (IPN) scaffolds’, that is, scaffolds formed from two or more polymers cross-linked together, made using Konjac glucomannan (a natural polymer from a plant source), polyvinyl alcohol and polycaprolactone were placed on the CAM. The IPN scaffolds were found to have angiogenic activity, with details on the thickness and quantity of blood vessels seen, although histology is often recommended to reaffirm findings.^115^

Biocompatibility of hydrophilic, porous, gelatin-polyvinylpyrrolidone (PVP) scaffolds was tested using the CAM assay by Mishra et al.^45^ Angiogenesis was not investigated or quantified in this study, with photographs taken at the time of material implantation and 3 days later, with no apparent indication of survival or normally developed chick numbers. It is reported that there was no effect on blood vessels compared with filter paper applied to each egg concurrently; therefore the material was deemed biocompatible due to lack of an obvious inflammatory response. However, it could be argued that a lack of any angiogenic response would limit the use of this material in further *in vivo* studies, as a lack of angiogenesis will critically affect the ability to repair large non-union defects.

## The creation of extracellular matrix and evaluation on the CAM

Extracellular matrix (ECM) materials have been shown to significantly enhance angiogenesis and ingrowth of blood vessels into a scaffold on the CAM, and decellularised or synthetic ECM is a burgeoning area of research to generate an ‘off the shelf’ product.^116^ ECM production requires cells to be present, differentiate and produce the surrounding matrix. Thus, the ECM can be created *in vitro* by cells seeded onto a scaffold prior to decellularisation, or with the addition of xenogenic cells to the construct *in vitro* prior to applying on the CAM, or by resident avian cells on the CAM invading the scaffold. The time taken for ECM to form can be affected by the cell numbers available, cell type and underlying scaffold material properties, which may mean the *ex vivo* organotypic culture of chick femurs may allow more time for potential ECM development. Due to the importance of the ECM, scaffolds with *in vitro* deposited, decellularised ECM have been developed based on the observation that ECM enhances angiogenesis.^69,116^ In the CAM model, Stro-4+ ovine BMSCs/bovine ECM (bECM) hydrogel with an electrospun PCL sheath was able to induce bone formation at an *ex vivo* chick femur fracture site, compared with the application of bECM hydrogel or blank defect alone.^58^ This led to translation of the scaffold into a tibial segmental critical defect study in sheep for further analysis.^58^

The use of scleral ossicles, in this case from chickens, was used as a naturally decellularised scaffold on the CAM and shown to induce an angiogenic response, likely due to the bone releasing growth factors.^74^ More recently, PCL scaffolds cultured with murine osteoblast/osteoclast-like cells and subsequently decellularised by freeze/thawing and DNase application, increased angiogenesis and displayed enhanced blood vessel penetration.^69^ Commercially available bone substitute (Bio-Gen) with hyaluronic acid resulted in osteoblastic differentiation of chorion cells, validated with immunohistochemistry, with a concurrent angiogenic response.^56^ Due to a lack of immunocompetence, cells can be used on the CAM and human adipose tissue-derived stem cells, seeded onto collagen coated bioglass scaffolds, led to a vascular response due to factors secreted by the cells.^105^ Conversely, 45S5 bioglass-derived glass-ceramic scaffolds were used by Vargas et al.^106^ on the *ex ovo* CAM at ED 10 but there was negligible angiogenic response. Interestingly, the authors observed a very poor survival rate in this study, although this was not discussed further.^106^ However, the chick bodies were deemed to have grown longer in length, possibly from mobilising calcium from the scaffolds as the *ex ovo* chicks become calcium deficient at ED 9 of incubation. Supplementing calcium by application of eggshell on an *ex ovo* CAM assay was found to contribute to skeletal growth and control the calcium transport mechanism and CBP activity.^117^

## Magnetic fields in bone tissue engineering

New methodologies have been combined with the CAM to develop angiogenic and bone tissue engineering approaches. Thus, the application of magnetic fields is gaining popularity in bone tissue engineering as a method of stimulating responsive particles and creating scaffolds. One approach has included the magnetic field stimulation of human mesenchymal stem cells labelled with a synthetic peptide linked to Wnt receptor, Frizzled, which resulted in enhanced bone mineralisation in a chick femur model when BMP-2 releasing microparticles were concurrently delivered.^118^ Magnetic pre-vascularised sheets layered together, using neodymium rod magnets, and constructed from HUVECs and adipose-derived stromal cells were observed to be osteogenic *in vitro* and angiogenic in the CAM, due to production of VEGF and BMP-2 from the construct, which may have exciting future applications in bone tissue engineering as co-culture of cell types is further explored and if the patient’s own cells could generate this scaffold on an ‘as needed’ basis with relative ease and low morbidity.^119^

## Use and evaluation of human bone development on the CAM

**Figure 3.**
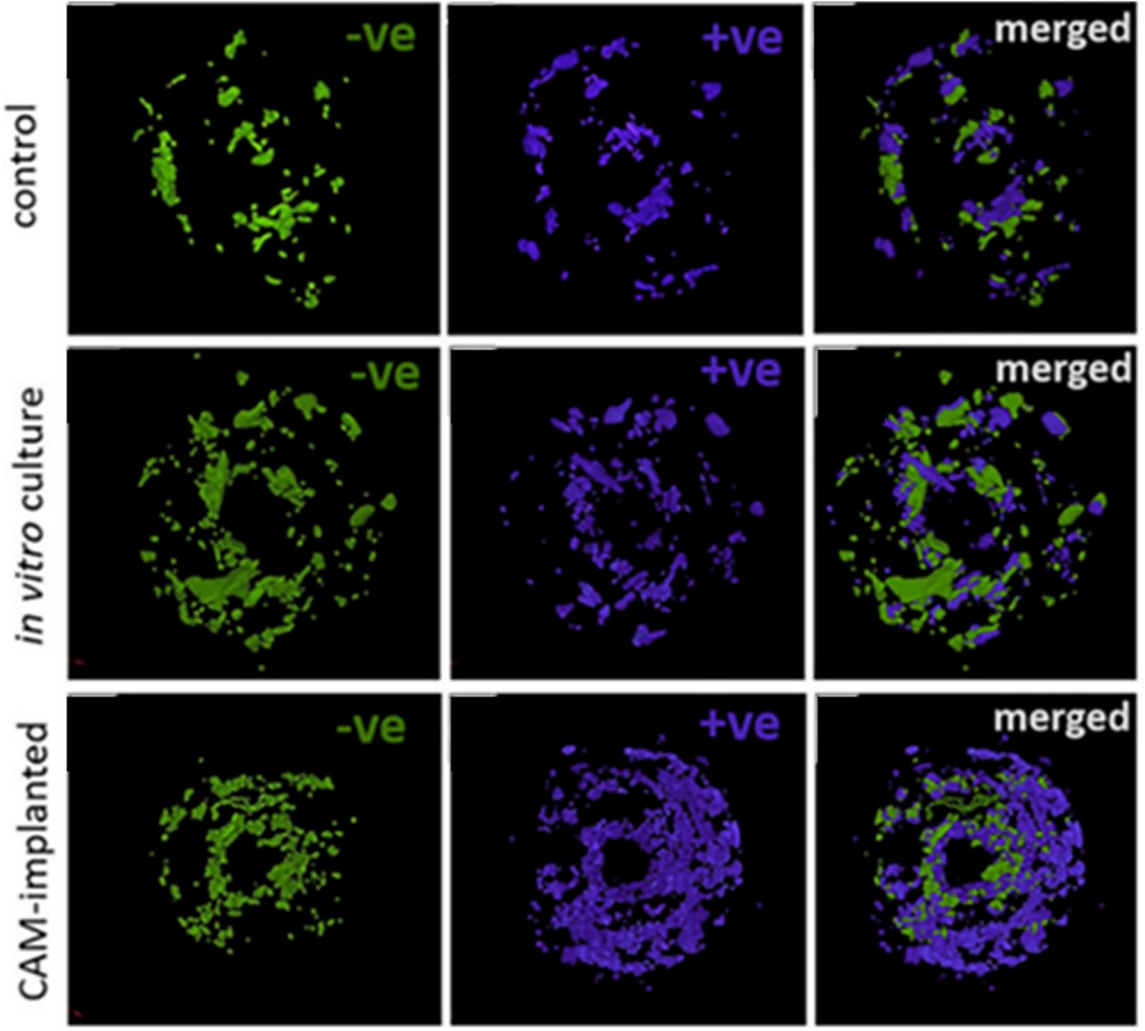
Bone cylinders extracted from human femoral heads pre-and post-incubation μCT scan images show areas of bone resorption (−ve) and bone deposition (+ve) after incubation on the CAM, *in vitro* culture or control (samples maintained at 4°C), followed by merging of the –ve and +ve images to view overall bone loss/gain within the bone cylinder in 3D (merged). *Source*: Figure reproduced and adapted from Moreno-Jiménez et al.^50^ with permission from *Journal of Tissue Engineering and Regenerative Medicine*.

In the pursuit of bone tissue engineering and the study of bone repair, cores of human bone trephined from femoral heads have been placed on the CAM to assess cell movement, integration and bone remodelling (Figure 3). Moreno-Jiménez et al.^51^ detailed approaches using chick embryos expressing green fluorescent protein to allow determination of cell source from bone core versus chick host. The cells from the chick were found to migrate to the acellular bone or material, for example, collagen sponge with BMP-2, to form bone, which could be analysed by microcomputed tomography (µCT) and histology.^50^

Allograft using decellularised, processed bone is an option to fill bone defects when autograft or other materials are not suitable; however, there is a high rate of failure to integrate due to lack of vascularisation.^120^ Holzmann et al.^120^ used the CAM to examine the clinical question of why there is a high rate of failure with this method. The authors observed that freezing of the bone had a negative effect on the angiogenic properties, compared with fresh bone, confirming that autograft is still optimal in bone tissue grafting. The use of xenograft transplants of bone and cartilage on the CAM was published in 1964 by Stephenson and Tomkins,^121^ with varying degrees of success dependent on species of tissue, age and conditions. Therefore, further studies on bone and cartilage development or regeneration should consider the CAM to create an *ex vivo* model.

Thus far, there are many options in bone tissue engineering which can be applied to the CAM, from growth factor solutions to encapsulated growth factors or the use of angiogenic compounds. The relative low cost of incubators and eggs is a significant attraction in the use of the CAM assay as well as the ready availability of chicken eggs, which are not subject to quarantine rules, the typical short time frame for studies and the advantages conferred within a 3Rs perspective, as detailed above. The aim is to use the scaffold as an osteoconductive platform, but also to link to or create a reservoir of osteoinductive substances to be released, differentiating cells down an osteogenic pathway during osteogenesis. The methods of the CAM assay are described and discussed followed by the *ex vivo* organotypic culture method used to assess the potential of osseointegration of the scaffolds prior to further *in vivo* studies.

## *Ex ovo* versus *in ovo* CAM assay

**Figure 4.**
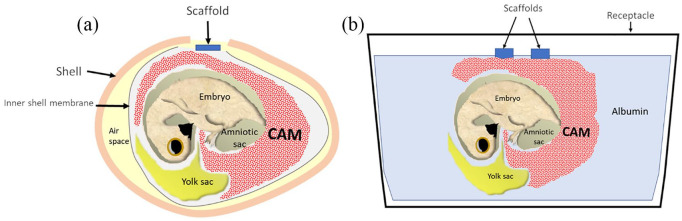
(a) Schematic illustration of the *in ovo* CAM assay with a defect in the eggshell/outer shell membrane (attached to shell) and inner shell membrane to allow application of the scaffold on the CAM. (b) Schematic illustration of the *ex ovo* CAM with the egg contents transferred to a sterile receptacle at ED 3 and the CAM develops, allowing multiple scaffolds to be applied.

The CAM assay can be performed using two methods: *in ovo* or *ex ovo. In ovo* describes the chick developing *within* the egg compared with *ex ovo* in which the egg contents are incubated *outwith* the egg (Figure 4). These methods have been extensively reviewed with the various differences within each method described, although there remains an absence of standard protocols for either CAM Assay.^122^ An *ex ovo* method is thoroughly described by Mangir et al.^4^ with over 80% survival reported using the reported methodology although only with experienced operators, while only 25% survive for beginners, making the *ex ovo* approach more difficult than the *in ovo* method.

The *ex ovo* method allows visualisation of the whole CAM from ED 3 and multiple materials^72^ can be applied to each CAM, reducing the chick numbers required.^123^ Opening the egg into a container at ED 3 of incubation is deemed optimal, as the CAM is not adherent and the yolk sac is less likely to burst.^123^ However, one issue remains, as when testing different growth factors or potentially harmful soluble materials, the presence of different growth factors or materials on a single CAM does not allow assessment of a material’s toxicity. Furthermore, if one factor, for example, BMP-2 is added to each scaffold, the collective dose of growth factor that the embryo is subjected to is unclear. Furthermore, the effects that a collective dose may have on development and subsequently any results generated are unclear.

Kohli and co-workers detailed another *ex ovo* method,^72^ in which the contents of the egg are suspended by sterile cling film over a glass filled with water, retained using a band around the top of the glass. This is similar to a method described in 1987, by De Jonge-Strobel et al.,^124^ and it should be noted that due to the lack of shell, skeletal formation is retarded, although osteoblasts and osteocytes were found to be normal. This cling film method provides survival rates of over 60%.^72^ It has been noted that embryos die due to the hard, flat material of the Petri dish, the increased surface tension and/or poor egg cracking technique, leading to rupture of the yolk membrane.^125^ Therefore, alternative *ex ovo* methods have been explored, such as application of a cup-CAM method where the egg resides within the bottom of an ice-cream cup to ensure that the embryo is not stretched, leading to 85%–90% survival rates.^125^ The cup-CAM method was developed to circumvent issues observed using the glass and cling film methods, with the egg contents observed to move and, in addition, sub-optimal depth parameters and scaffolds ‘sinking’.^125^ A cubic artificial eggshell with a defined pattern to guide blood vessels from the CAM has also been developed; however, widespread use of this model does not, at present, appear likely due to low survival rates and construction of the cube.^126^

*In ovo*, sterility is often considered an issue, although *ex ovo* culture, with the obvious absence of an eggshell for protection, typically results in the use of prophylactic antibiotics. Many research papers and methodology texts describe the removal of albumin from the egg^46^ or drilling a hole^92^ to ‘drop’ the CAM away from the inner shell membrane, as damage to the CAM at implantation can lead to increased or decreased angiogenesis and interfere with the results.^18^ Removal of albumin can contribute to poor survival and, as a consequence, alternative methods are sought which are less invasive, including creating a vacuum through a small pin hole^127,128^ or use of a diaphanoscope to illuminate the egg to avoid damaging the embryo.^46^ Other authors provide less clear instructions or indeed outdated/contraindicated instructions, including cleaning the eggs with ethanol,^129^ which has been reported to reduce survival rates.^46,123^ A study by Kivrak Pfiffner et al.^130^ indicated poor survival rate of 30%–40%, which would not produce a statistically valid output with comparative treatments and thus compromised the concept of reduction and refinement. It should be noted that cleaning of eggs with water is not recommended, given the increased risk of infection, although, again publications indicating such an approach have been seen in recent times.^46,125^ Egg washing for consumption is not permitted in the United Kingdom, given the porous nature of the eggshell and thus contaminants transfer into the egg. Thus, wiping the egg with a paper towel is deemed sufficient to remove gross contamination. In addition, the formation of a large window can be a risk factor for infection or eggshell dust on the CAM.^125^ In our laboratory, the eggs are not cleaned prior to incubation, but at the time of material implantation, a paper towel sprayed with high-level laboratory disinfectant is used to wipe the small area where the window will be immediately defined. No detrimental issues with survival rates (typically 85%–100%, with chicks failing to form rather than dying *in ovo*) have been seen with this method, and such an approach ensures that the edges of the window are sterile (protecting scaffolds if any contact is made with the shell while the scaffold is placed on the CAM). Eggshell dust can prove to be an irritant for the CAM;^72^ however, if the hard shell is removed, leaving the white inner shell membrane intact, as in our approach, then the dust can be removed before the CAM is exposed. Critically, for *in ovo* incubation, antibiotic or antimycotic^72^ solutions are not necessary compared with *ex ovo* methods and eggs can be incubated ‘end on’ or ‘side on’ as described in articles and reviews.^51,122^

## Current experimental methodology

The *in ovo* method used within our laboratory for biomaterial testing is described below.

### Materials and reagents

- Egg incubators with adjustable rotation (Hatchmaster incubator, Brinsea, UK);
- Deionised/distilled water (DDW) for incubator tray to maintain humidity (60%);
- Thermometer and hygrometer inside incubator;
- Fertilised chicken eggs (*Gallus gallus domesticus*);
- Torch for candling eggs;
- Class II laminar flow cabinet;
- Small egg box with points inside cut off;
- High-level laboratory disinfectant and paper towels;
- No. 10 scalpel blade or small saw blades or battery-powered engraving pen;
- Sterile forceps for opening shell/inner shell membrane;
- Sterile forceps for handling materials;
- Sterile materials to be implanted – appropriate size and weight;
- Parafilm squares soaked in 70% ethanol and washed in sterile 1× PBS;
- Phosphate buffered saline (PBS);
- Autoclave tape labelled with ‘code’ for implanted materials;
- NB sterilisation of instruments and the egg box is performed by autoclaving as ethanol is not sporicidal and may ‘fix’ proteins to the equipment.

### Experimental numbers and experimental plans

Typically, an *n* = 6 for each condition (to allow predominantly for non-developing eggs) is used within our group. The time of year can be a factor with March to May optimal, as fertility declines in the winter months.^131^ One or two eggs are typically used as ‘non-implant controls’ to determine normal chick development (no intervention applied to the CAM, but a window is created).

Hens (*Gallus gallus domesticus*) eggs are typically received at 12–18°C to prevent chick development in transport, and gradually reach room temperature to prevent condensation within the egg, prior to placement in humidified (60%), 37°C incubator(s). High environmental temperatures can therefore affect egg development and viability if used in summer. The eggs are incubated for 10 days horizontally on a rotating pattern (1-h scheduled rotation) at 37°C and 60% humidity. Alternatively, the eggs can be stored in stasis at 12–14°C in a basic incubator until the experiment is ready to begin. Do not store the eggs longer than 7 days, as viability will be reduced.

### Implantation of materials

To limit handling, the risk of infection and time taken, the eggs are opened only once at implantation of materials. There is no major benefit of opening the eggs at an earlier time point of ED 3 to detach the shell membrane from the CAM with this method. Importantly, albumin is not removed, the CAM is not imaged and therefore no manipulation is necessary. The day of implantation varies, but ED 10 of incubation is commonly when scaffold materials will be implanted, as the CAM is more developed to support the weight and size of the scaffolds with rapid angiogenesis and growth having occurred. In addition, this provides a 7–10 day window for the incubation of test samples compared with use later at EDs 12–13, which would reduce available incubation time due to hatching at ED 21. It has been found that after ED 15, a non-specific inflammatory reaction can occur and therefore control eggs are essential to compare results.^6^ When windowing the eggs and removing the white shell membrane, it is important not to damage the CAM or induce haemorrhage if at all possible, due to the delicate structure of the CAM tearing with the result that the scaffold could disappear into the egg rather than sitting on the CAM. Such an event will result in lack of integration or chick death:^122,132^

**Figure 5.**
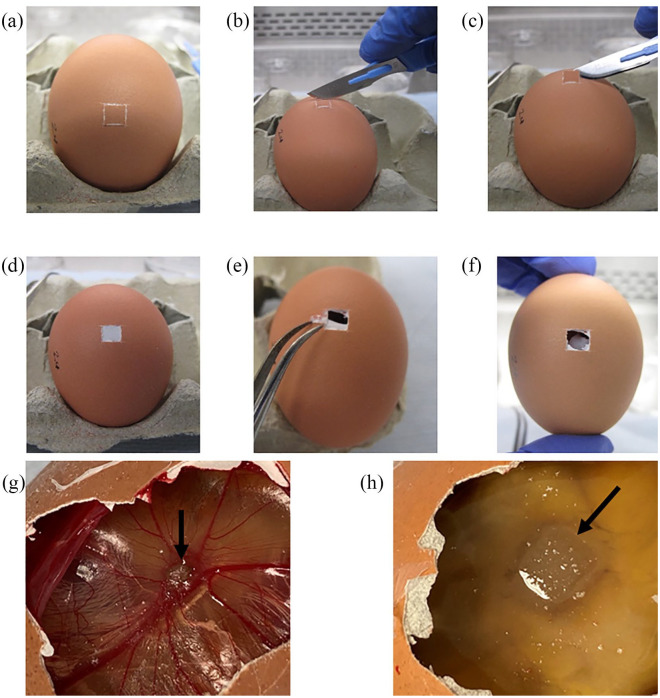
(a) Score as small a window as possible to fit the scaffold/sample through with a scalpel blade. (b) Continue moving the long edge of the blade back and forth across the egg. (c) Tilt the blade and use the long edge to remove the eggshell. (d) The white inner shell membrane is visible. (e) Pierce the membrane and peel away. (f) Collagen sponge within the egg on the CAM. (g) Collagen sponge soaked in 140-ng BMP-2 (black arrow) at ED 18 with an angiogenic response seen. (h) Collagen sponge soaked in 140-ng BMP-2 (black arrow) at ED 18, but the chick has not formed.

- Laminar flow cabinet is cleaned with high-level laboratory disinfectant to remove all dust and to create a sterile environment.
- Appropriate laboratory gown/coat is worn and impermeable gloves used for all work undertaken in the laminar flow cabinet.
- The laminar flow cabinet should be set up with a sterile egg box to hold the egg and sterile forceps, a No. 10 scalpel blade, parafilm squares (2 cm^2^) in 70% ethanol and autoclave tape to secure the parafilm.
- Eggs should be candled to check viability and to confirm a dark shadow (embryo uppermost) with the egg placed in a horizontal position on the egg box and the narrower section away from the operative.
- A ‘palm grip’ is used on the scalpel handle to create a 0.5 cm × 0.5 cm window (slightly off centre towards the wider end of the egg) using a No. 10 scalpel blade. The four sides of a square should be created to provide a guide (Figure 5(a) and (b)). Note that the cutting may become faster as the blade becomes duller. The eggshell will feel ‘gritty’ and it may become difficult to move the blade across the egg as the shell becomes thinner.
- Remove the piece of shell with the blade at a 45° angle (Figure 5(c)).
- The white inner shell membrane should now be visible (Figure 5(d)).
- Make a small perforation in one corner of this membrane using sharp, narrow, curved forceps and gently peel off the white inner shell membrane to reveal the CAM below (Figure 5(e)). Note that it is easy to cut the CAM at this point and minor bleeding to occur, but the CAM should remain intact for materials to be placed upon.
- Add the material to the CAM through the window created (Figure 5(f)).
- Parafilm (soaked in 70% ethanol, rinsed in PBS then drip dry) is typically used to cover the window by gentle stretching – do not overstretch as the incubator heat will cause the parafilm to shear, facilitating desiccation of the CAM. Ideally, hold the centre of the parafilm square and stretch the edges only over the window created.
- Apply labelled tape to both sides of the parafilm, parallel to sides of the egg, to hold in place.
- Place the eggs horizontally within an incubator for 8 days at 37°C and 60% humidity without rotation.

### Analysis of results

**Figure 6.**
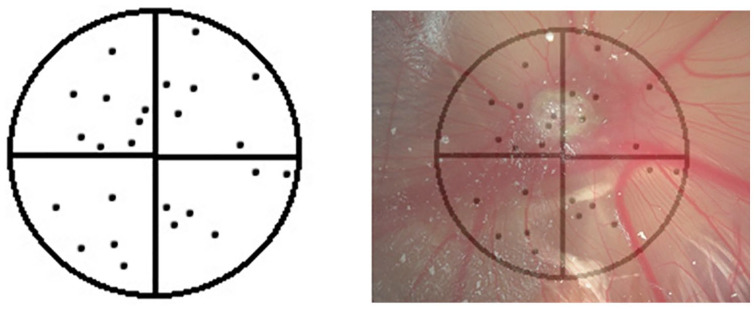
Example drawing of a Chalkley graticule eyepiece showing the layout of dots to align over blood vessels, allowing counting to quantify the angiogenic response to materials, such as BMP-2 soaked collagen sponge shown.

- At ED 18 of incubation, the tape and parafilm are removed and the ‘window’ opened using wide, flat tipped forceps (ensure shell dust does not enter the egg).
- The scaffold is imaged using a stereomicroscope and digital camera (Figure 5(g)).
- Biocompatibility is assessed by counting live, viable and developed chicks and any dead/deformed chicks.
- Quantification of angiogenesis can be performed using the Chalkley score method (Figure 6).
- The material and surrounding CAM tissue (0.5 cm margin) can be harvested for histology.

In our laboratory, photographs of each egg are taken using a stereomicroscope with a digital camera for records. Biocompatibility is assessed using chick viability and absence of developmental issues, potentially caused by growth factor or drug use. The number of live, abnormal or dead chicks and non-formed chicks (Figure 5(h)) are determined. The blood vessels surrounding the material on the CAM are assessed using the Chalkley score method. A Chalkley eyepiece graticule (Figure 6) is inserted into the eyepiece of a stereomicroscope and the scaffolds viewed at a standardised magnification. The centre of the cross of the eyepiece is positioned over or at the edge of the scaffold with the greatest angiogenic response and rotated to align as many of the 25 dots of the Chalkley eyepiece graticle as possible over blood vessels. Three separate counts are taken and the average score is calculated for each egg. Thereafter, the sample is removed from the CAM, with 0.5–1 cm of surrounding CAM tissue, using sharp scissors and forceps. The chick is euthanised according to specific UK Home Office Guidelines. The tissue is placed into 2 mL of 4% paraformaldehyde (PFA) in a 24-well plate for 72 h at +4^o^C followed by exchange of PFA for 70% ethanol if storing for longer periods. Processing of the tissue, embedding and subsequent histology can follow.

## Experimental approaches to quantify angiogenesis and assess biocompatibility

### Manual and computer-aided quantification

Quantification of angiogenesis varies significantly between studies; however, the experimental techniques used to visualise cell and tissue responses are becoming more robust. Selection of areas at random on the CAM is employed when a diffuse substance has been applied to the CAM. The blood vessel length, width^84^ and branching^72,110^ are able to be measured.^84^ However, this method is subjective and, typically considers angiogenesis in direct contact with the implanted material or on the surface.^40^ Older methods still in use today, typically detail defining a radius around the scaffold or implantation site and counting blood vessels.^96,100,133^ The Chalkley scoring method provides an estimate of blood vessels, rather than a real counted value of vessels, as three counts of the dots aligned over blood vessels are taken and averaged to give the Chalkley count to reduce the variability in readings.^134^ This method has been found to give consistent results when used for histological tissue samples in oncology research.^135,136^ Due to the smooth nature of the CAM, it could be speculated that the Chalkley method is reliable for hydrogels and materials which conform to and contact the surface of the CAM, whereas angular 3D constructs may struggle to provide uniform contact and therefore lead to irregularly surrounding blood vessel formation. However, the scaffold usually integrates with the tissue and can be further assessed histologically. Taking the average of three or more Chalkley score measurements should give a more accurate account of angiogenesis. Blinding of the observer(s) can limit bias in the study.^137^ Other enhancements to aid vessel numeration include injecting an agent under the CAM from a distance to the scaffold, such as hand cream, providing a white background for contrast.^4^ Software such as Photoshop,^4^ Image J,^4,69,72,87^ Angiotool^4,69^ or AngioQuant (v1.33)^115^ can be used to delineate the area of analysis, highlight or alter the image to demarcate the blood vessels more clearly, or perform automatic counting. Computer simulations have been investigated to automate the Chalkley counting process, making analysis more rapid and, critically, were observed to be as reliable as manual counting.^134^ Measurement of blood vessels in relation to scaffold size is an option, given some scaffolds will reduce in size over time, for example, collagen sponge, allowing computer analysis and measurement of vascular density.^72^ Fixation of the CAM with PFA, dissecting out the scaffold and imaging from below, to enable penetrating blood vessel visualisation, is another reported method.^72^

### Histology and immunohistochemistry

Histology provides a key tool to assess the infiltration of blood vessels into a construct applied on the CAM. The CAM is made of three layers – the endoderm, ectoderm and mesoderm.^40^ Fixation of the scaffolds in situ limits bleeding from small vessels when removed from the CAM.^72^ Critically, inflammatory angiogenesis must be distinguished from an angiogenic response due to the material analysed at later time points, by histology and immunohistochemistry^40^ as monocytes and inflammatory-like cells are active in the CAM.^138^ Bai et al.^92^ looked at interleukin-10, interleukin-12 and nitric oxide release to determine macrophage activation in response to their growth factor releasing scaffolds. Using histological sections to count blood vessels may underestimate results, as used by Strassburg et al.,^139^ to quantify angiogenesis after using fibrin matrix with adipose-derived stem cells and endothelial cells on the CAM using a modified ‘cylinder CAM assay’. Empty blood vessels may be missed, despite a computer program being used in analysis.^139^ The reported ‘modified cylinder CAM assay’ method uses a plastic cylinder of appropriate height which sits on the surface of the CAM, and the opposite end is held by a lipped edge supported by the eggshell.^140^ This construct enables cells within a fibrin matrix to be deposited within the cylinder and a cap to cover the cylinder closing the egg.^140^ This cylinder ensures that the tested material stays in place on the CAM, prevents drying of the CAM and evaporation of media added to the cells applied within the cylinder.^140^ It is important to note the application of media over the CAM surface can affect chick survival due to interference with the gas exchange function of the CAM.^140^ However, this method does not appear to have gained widespread use, most likely due to the requirement for plastic cylinders of different depths to be available to be placed exactly in the egg between the CAM and the shell. Studies using materials which require containment on the CAM can be placed into inert rings, such as those made of silicon.^56,119^

Within the bone engineering sphere, immunohistochemistry using antibodies for RUNX2 (transcription factor directing osteogenesis and a marker of osteoblast differentiation), SPARC (a marker for bone glycoprotein) and BMP-4 (required for osteosynthesis) have been used to assess cellular differentiation.^56^ Other antigens, Cathepsin K (signifies osteoclast resorption) and SOX9 (transcription factor directing chondrogenesis), were included in a human bone core experiment, which found that the avian cells of green-fluorescent protein (GFP) transgenic chick embryos invaded the human bone core and were responsible for bone turnover.^50^ Recently, Petrovova et al.^40^ used quail eggs as the endothelium of the quail CAM expresses a unique marker QH1. This approach prevents the need for genetically modified chick embryos or exogenous application of fluorescent cell labelling. QH1 can be visualised by a specific antibody, allowing angiogenesis to be visualised more clearly. In another study, mouse blood vessel endothelial cells were stained using anti-CD31 antibody to differentiate from quail endothelial cells and it was found that a chimeric vascular network formed within differentiated tissue.^141^ Therefore, the ability to determine grafted cells from avian host cells is important for determining cellular communication and differentiation, active cell invasion and attachment to materials. Cells can be pre-labelled with fluorescent markers, provided the dye lasts for a sufficient time period, prior to seeding on materials for use on the CAM. GFP transgenic chicks or quail eggs can be used to label the host cells, or immunohistochemistry targeting various cell types can be used.^142^ Molecular analysis of specific gene expression in tissues related to angiogenesis is mentioned by Vargas et al.,^143^ although such an approach does not appear to be commonly reported or discussed in the recent literature.

### 3D imaging of the CAM

**Figure 7.**
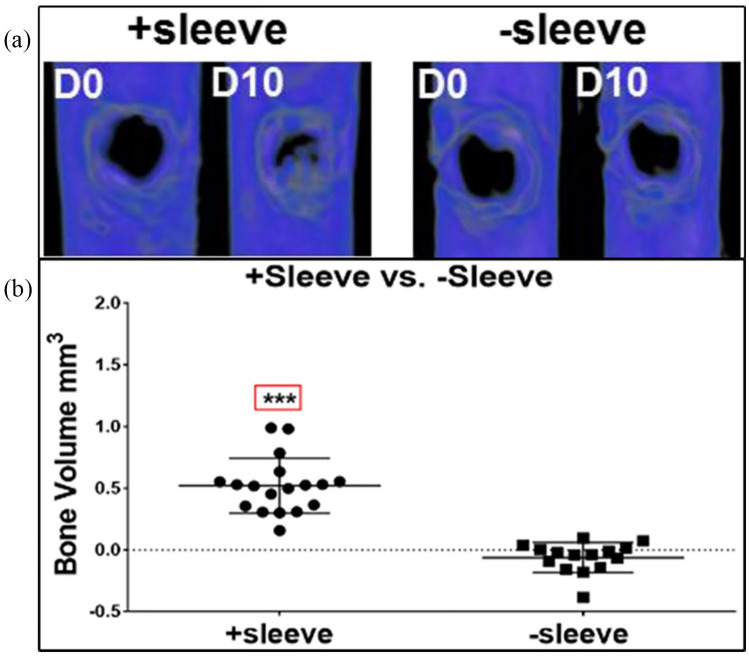
(a) μCT scan images of femur defects at days 0 and 10, with or without a surrounding human placental vessel sleeve, showing increased bone formation with the sleeve. (b) μCT data analysis of increase in bone volume (BV) change at day 10 of culture in femur defects with cell pellet implant within a sleeve or without a sleeve covering. Treatment group with a sleeve demonstrated a significant increase in BV using Student’s *t*-test analysis. Data are presented as mean ± SD. Significance set at ***p ⩽ 0.0001. *Source*: Figure adapted and reproduced from Inglis et al.^148^ with permission from *Advanced Healthcare Materials Journal*.

Woloszyk et al.^144^ describe a method of 3D imaging blood vessels using µCT following perfusion with ‘MicroFil’ – a radiopaque substance, which is perfused into the vasculature prior to fixation. This allows blood vessels within scaffolds to be observed, rather than quantification of vessels in the surrounding area, and offers additional information on the effects of material choice and porosity. This technique also permits immunohistochemistry post tomography. µCT is a sensitive method to assess bone formation or remodelling and has been used to calculate the overall bone gain/loss of human femoral bone cores^50,51^ (Figure 3), chick femurs^145–148^ (Figure 7) and could be used to assess bone formation within scaffolds on the CAM.^149^

Magnetic resonance imaging (MRI) to measure vascular perfusion of scaffolds or materials on the CAM has also been described.^116,130,149^ Alternatively, a marker that targets hydroxyapatite was used to highlight mineralised deposits on scaffolds.^150^ An advantage of MRI would be the ability to non-invasively assess functional vasculature formed at the surface, middle or tissue interface of the scaffold;^116,149^ however, concerns with this method include the availability of an MRI machine and the reported use of ketamine to sedate the embryo during imaging. At the time of writing, no studies could be found reporting the change, if any, in vascular resistance to topical ketamine on the CAM; however, the change in vascular resistance should be equal in all test subjects if a consistent method is used, allowing comparisons between materials on the CAM to be made.^151^ The injection of contrast agent in this study also necessitated the use of a surgical microscope, which may not be accessible for all researchers. Woloszyk et al.^149^ combined MRI *in ovo* using gadolinium-based contrast agent and µCT post-perfusion with ‘Microfil’ to provide an overall 3D assessment of functional blood flow and quantification of vascularisation. Using a material like cortical bone (Optimaix^™^), and another similar to cancellous bone (DegraPol^®^), the method was validated, and the functional blood flow calculated by MRI correlated to total vessel volume determined by µCT. Histology was used in this study to give further detail at the cellular level, allowing a complete assessment of the interaction and angiogenic response of the CAM to the scaffolds. Medetomidine was used by Woloszyk et al.^149^ to sedate the chicks *in ovo* for MRI in this study, as it has been found by Waschkies et al.^151^ that medetomidine (0.3 mg/kg) applied topically to the CAM was more effective than ketamine/midazolam or thiopental at reducing movement of the chick, and therefore motion artefacts, for 30 min which was sufficient time to generate MRI images safely.

## *Ex vivo* organotypic culture

Not only can the CAM be used for research development and evaluation, but bone from the chick can be used to assess bone tissue engineering strategies in an *ex vivo* organotypic culture method (see detailed review in Smith et al.).^142^ The femur is commonly used, as it is relatively large and shows endochondral ossification. ED 11 of incubation was observed to be the optimal time point for investigation of bone formation, as skeletal differentiation and mineralisation commence at this time.^152^ The method using ED 11 femora allows the incubation and euthanasia of chicks to be completed prior to ED 14, therefore no personal licence is required to perform this assay; however, training in appropriate euthanasia methods is still required. Interestingly, using older chicks may mimic *in vivo* bone healing in a more mature bone environment.^153^ There are no legislative controls in the United Kingdom regarding the use of biological material from deceased animals; however, ethical review and notification may be a requirement of each different university or research facility and therefore this should be verified prior to starting any *in vivo* or *ex vivo* experimentation. The long bones can be harvested from the chick at various time points of incubation, depending on the study, but it would be prudent to consider using the bones of chicks euthanised at the end of CAM assays, which had no material applied or were negative controls to ensure normal chick bones could be used and recycled from valuable biological material. In addition, this method would allow the CAM assay to assess angiogenic potential of materials prior to then setting up organotypic culture to assess the ability of these materials in a highly cellular, osteogenic niche. Such an approach enables further assessment prior to mammalian *in vivo* models. However, the limitations of this method include the lack of a blood supply, thereby reducing nutrient and oxygen delivery to cells, and therefore a 10-day culture period is adequate to study changes.^152^ The lack of blood supply also limits immune cell and osteoclast presence at the defect; however, this may be negated by use of the *ex vivo* femur culture on the surface of the *in vivo* CAM assay.^153,154^ A lack of mechanical forces in this model affects tissue formation, however, *in vitro* bioreactors could potentially be used to imitate forces applied.^154^ In addition, a femoral defect provides an ideal test bed for materials/scaffolds, as the femurs can be transected or a drill hole defect created, and materials (malleable, e.g., hydrogel scaffolds) inserted.

### Materials and reagents

- Class II laminar flow cabinet;
- Fertilised hen eggs (*Gallus gallus domesticus*), ideally at ED 11 of development;
- Semi-porous (0.4-µm pore size, 30-mm diameter) polycarbonate membrane well inserts;
- 6-well plate;
- Malleable/gelatinous material to be tested in defect model (diaphyseal defect, drill hole, or cortical defect);
- Basal medium of α-minimum essential medium (α-MEM) and ascorbic acid-2-phosphate (100 µM), for example;
- Antibiotics (penicillin (100 U/mL), streptomycin (100 µg/mL) can be added if there are concerns regarding sterility of procedure or samples, but are avoided if possible;
- Culture medium containing test substances (for example, growth factors, vitamin D_3_) is used as appropriate;
- Foetal calf serum may be added to positive control samples to encourage bone growth;
- Three sterile petri dishes;
- Sterile phosphate buffered saline (PBS);
- Sterile instruments for dissection – forceps, scalpel blade, drill bit.

### Method for chick femur culture

**Figure 8.**
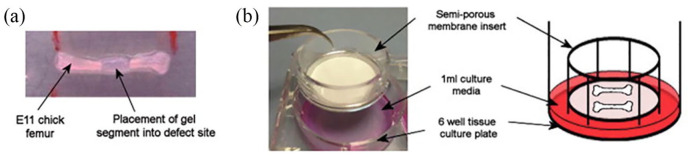
(a) ED 11 chick femur segmental diaphyseal defect model with gel within the defect. (b) *Ex vivo* organotypic culture set-up using a semi-porous membrane insert within a culture plate well and appropriate media to culture the bone at the liquid–gas interface. Figure adapted and reproduced from Smith et al.^145^ with permission from Acta Biomaterialia.

**Figure 9.**
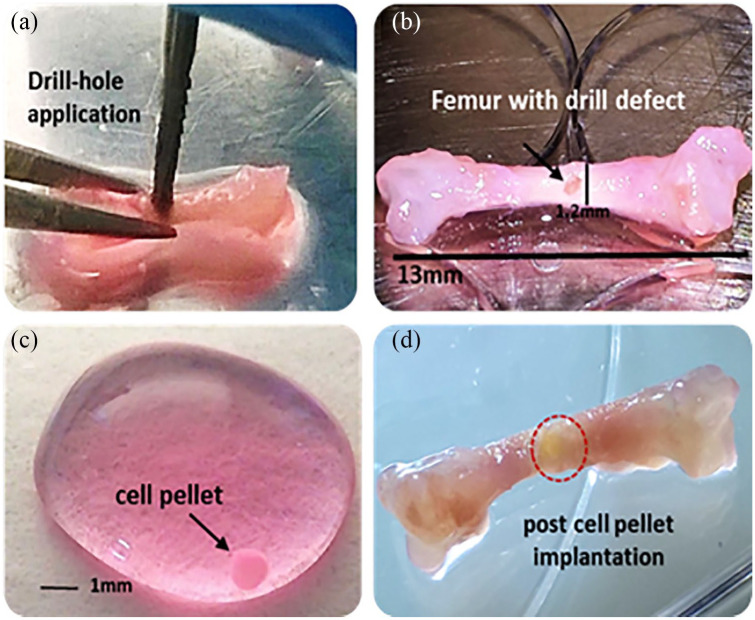
(a) Bone defect model using a drill bit (b) to create a mid-diaphyseal circular hole for implantation of cells/materials into an ED 18 chick femur. (c) A cell pellet is cultured (d) for implantation into defect. *Source*: Figure adapted and reproduced from Inglis et al.^146,148^ with permission from FASEB J and Advanced Healthcare Materials.

- Laminar flow cabinet is cleaned with high-level laboratory disinfectant to remove all dust and to create a sterile environment;
- Appropriate laboratory gown/coat is worn and impermeable gloves used for all work undertaken in the laminar flow cabinet;
- Crack open the egg on the side of a Petri dish and empty the contents of the egg into the dish;
- Euthanise the chick by an appropriate method, that is, decapitation;
- Remove the chick limb by dissecting from the body at the hip joint, taking care not to remove the delicate cartilaginous femoral head and place in a sterile Petri dish;
- Remove the soft tissues from the femur, taking care to leave the cartilage at either end of the femur and the periosteum intact – this can be done most easily using non-powdered sterile gloves by hand rather than sharp dissection; however sterility is essential, and the femur must be kept as intact as possible;
- Options are to transect the mid-femur to create a diaphyseal defect model, create a hole using a drill bit or cut the diaphysis on one side only to create a hollowed out, mono-cortical defect model which can hold a larger volume of test material (Figures 8(a) and 9);
- Rinse the femur in PBS in a sterile petri dish if there is any adherent tissue material;
- Implant the scaffold material/cells/pellet/gel into the defect.

*Ex vivo* method

- Put 1 mL of media in to each well of a 6-well plate;
- Place the membrane well inserts into each well;
- Place up to three femurs in each well at the liquid–gas interface (Figure 8(b));
- Culture for 10 days at 37°C in air at 5% CO_2_ with media changes every 24 h;
- At the end of the culture period, rinse the samples in PBS and fix the samples in 4% PFA prior to processing for histology or imaging by µCT;
- Control femurs should be used, either fixed immediately to compare size and structure to cultured femurs or with a defect omitting the insertion of a test material, that is, an ‘empty’ control.

*Ex vivo*/CAM hybrid method

**Figure 10.**
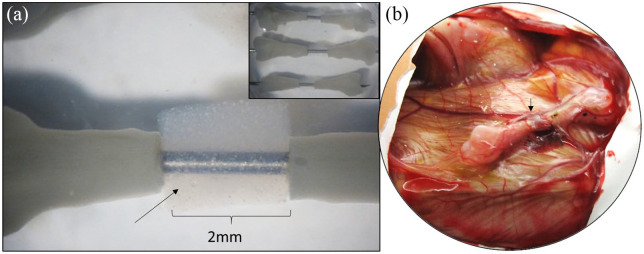
(a) ED 18 chick segmental femur defect (2 mm) with implanted bone extracellular matrix hydrogel (arrows) with BMP-2 encapsulated microparticles, three identical femur defects and hydrogel (inset). (b) Implanted bone defect and hydrogel after 8 days’ incubation in the CAM. All egg CAM procedures were carried out in accordance with the guidelines and regulations stipulated in the Animals (Scientific Procedures) Act, UK 1986 and under Home Office Project licence (PPL 30/2762).

- An alternative to the *ex vivo* culture method involves applying the chick femur ± test material to the CAM of additional chick eggs as per the method described for the CAM assay^58^ (Figure 10).

### Examples of *ex vivo* organotypic studies for bone tissue engineering

Alginate/bovine decellularised and demineralised ECM scaffolds with growth factors VEGF, transforming growth factor β_3_ (TGF-β_3_) and BMP-2 were applied to a 2-mm segmental chick femur defect model and cultured for 10 days. The VEGF and TGF-β_3_ generated a tissue with chondrogenic phenotype, whereas BMP-2 created a more osteogenic phenotype and repair evident upon histological staining.^145^ Further expanding on this work, Smith et al.^153^ found that TGF-β_3_ in combination with BMP-2 and Stro-1+ human bone marrow stromal cells (HBMSCs) generated the most significant effect on bone formation. Other factors known to be important in bone repair can also be studied using the *ex vivo* organotypic culture system, for example, vitamin D_3_, parathyroid hormone and parathyroid-hormone-related protein.^147,154^ Such *ex vivo* approaches provide a stepping-stone between factors added to culture media *in vitro* to *ex vivo* organotypic models, to potential use on the *ex vivo* CAM, to larger *in vivo* animal models.^147^ Furthermore, the culture and growth of chick femurs *ex vivo* also reduces preclinical animal testing by validating proof of concept prior to any further trials.^146^ Initial work by Inglis et al.^155^ used VEGF (100 ng/mL) in the culture media of an organotypic cultured femur model and found increased bone formation parameters and CD31 immunohistochemical staining (indicating endothelial cells), affirming the role of VEGF in osteogenesis and angiogenesis. Cell pellets can be inserted within a diaphyseal drill hole defect, as illustrated in Figure 9. Recently, Inglis et al.^148^ used human decellularised placental vessel sleeves alone or re-cellularised with HUVECs on the CAM from EDs 10 to 18 of incubation to assess biocompatibility. The authors reported successful integration and 100% chick survival. Further to this, ED 18 chick femurs with a drill defect in the diaphysis were cultured in organotypic culture in a well plate within a sleeve with or without a HUVEC pellet. The presence of the placental sleeve significantly increased bone formation, regardless of whether the HUVEC pellet was present, confirming the importance of a scaffold rich in growth factors to induce bone healing. HBMSCs combined with HUVECs in a 1:1 ratio co-culture spheroid was inserted into a chick diaphyseal defect and surprisingly the HBMSC and HUVEC pellets cultured separately had a more significant increase in bone formation than in co-culture.^146^ Another example of applications of the *ex vivo* use of chick femurs is shown in Figure 10. ED 18 femurs can be employed as a segmental defect model by implantation of a biomaterial in the defect site fixated with a 28 gauge needle and implanted into the CAM model for 7–10 days (Figure 10(a) and (b)). Organotypic culture has also used bone from other species, for example, rat, cut into thin (300 µm) sections and cultured for up to 21 days, which allows nutrition of the tissue for longer than a thicker 3D tissue to assess *ex vivo* bone development.^156^ Therefore, bone taken from rodents used in other *in vivo* studies could be recycled for *ex vivo* experiments to compare any differences seen in avian and rodent biology, for example, growth factor dose required for vascularisation/osteogenesis.

### Quantification and analysis of tissue formation

**Figure 11.**
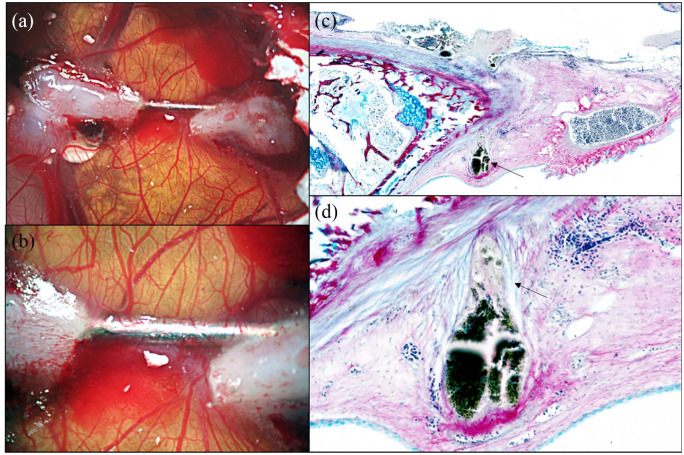
(a) ED 18 chick femur defect fixated with a 28-G needle and implanted into the CAM (8 days). (b) Note implanted needle with CAM blood vessels encapsulating it. (c) Alcian blue/Sirius red histology staining of the bone defect, for proteoglycan and collagen matrix visualisation, with the encapsulated CAM. (d) High-power image depicting large CAM blood vessel migrating towards the bone (arrows). All egg CAM procedures were carried out in accordance with the guidelines and regulations stipulated in the Animals (Scientific Procedures) Act, UK 1986 and under Home Office Project licence (PPL 30/2762).

The *ex vivo* organotypic culture method allows quantification of bone ECM formation and bone defect healing via imaging methods such at µCT and detailed examination of the tissue and cell types by histological methods.^152^ Scanning electron microscopy can be used to assess cell adhesion and morphology on constucts.^157^ µCT allows quantification of femur length, bone volume, trabecular number, thickness and separation to compare materials with the controls^145,153^ (Figure 7). Similar to the CAM assay tissue samples, histological stains used include Sirius red to demarcate collagen formation, Alcian blue to illustrate proteoglycans or Goldner’s trichrome for calcified, mature and immature bone matrix^148^ (Figure 11). Von Kossa staining illustrates areas of mineralisation within the femur or defect, while specific histological stains such as those for collagen type I/II or surface marker selected cells (e.g., Stro-1+) can be used.^147^ In-situ hybridisation using labelled probes for specific DNA sequences, molecular methods such as real time quantitative PCR and western blot have also been used to identify cell types, gene expression and protein production in other *ex vivo* tissue models.^46,158,159^

## Conclusion

Advances in bone tissue engineering will remain limited in the absence of a clear pathway from *in vitro* research to human clinical trials. A wealth of research indicates a material similar or based on the components of bone, for example, calcium based, or biocompatible, biodegradable polymers with a porosity suitable for angiogenesis and electrostatic charge which attracts cells, will find application in bone repair. Furthermore, while our understanding of the orchestration and role of growth factors in skeletal development and repair is evolving, identification of a material which supports the cells generating these growth factors endogenously, would limit concerns about the concentration to be used and the potential side effects. Much of the recent research has predominantly detailed the *in vitro* testing and validation of materials and growth factors prior to use in the CAM, which is prudent. Critically, the CAM assay provides important additional information to inform progression to preclinical *in vivo* models. There is no doubt that *in vivo* experiments provide an essential bridge to the gap between *in vitro* findings and clinical application of biomedical devices, pharmaceuticals and materials. Therefore, the CAM assay could potentially reduce the poor correlation seen between *in vitro* findings and those from *in vivo* models, as a prior ‘screening’ model.^160^ Current evidence, and our perspective, is that the CAM assay provides a rapid, cost-effective, versatile, robust and reproducible system, to assess materials prior to evaluation within an animal model. However, the method employed must be carefully considered. Importantly, standardisation of technique across the field will allow greater impact, conclusions and comparisons to be drawn, and potentially transform materials research in bone tissue engineering.

A database of materials used in the CAM and *in vivo* would be useful to assess what materials or substances have been applied, and the outcome of such experiments to prevent repetition, reduce wastage in time/money/research effort as well as allow refinement of promising materials to progress towards clinical translation. The ARRIVE guidelines provide a 20-part checklist of the minimum information required to be reported by groups using animals in research. ARRIVE guidelines are essential to help overcome issues in science such as reproducibility, reducing bias and the correct use of statistical methods of analysis.^66,161^ The ARRIVE guidelines have been used to create a database of mouse phenotyping data and could be applied to a large *in vivo* pharmacological assay results database.^162–164^ While a useful resource, a bone tissue engineering database would require to be structured and rely on standardised procedures to limit the variation in protocols; however, this may allow the optimal ‘evidence based’ methods to be used for future *in vivo* experiments.^163^ While journals of negative results have been published in the past, a similar resource that captured what may be perceived as negative findings could prove useful in supporting the formulation of new ideas/materials/approaches for future work. The relatively simple CAM assay and *ex vivo* organotypic culture protocol outlined in this review, completed early in the incubation period (to ED 14), allows CAM use in the absence of licensing and regulation; however, as emphasised, welfare, the method of implantation and analysis of results must be carefully considered to gain the maximal information from each experiment. The increasing use of the CAM assay and *ex vivo* organotypic culture in the field of bone tissue engineering is predicted, given the urgent clinical musculoskeletal requirements to be addressed in an increasingly ageing population. In summary, the CAM assay, far from an archaic, redolent assay, provides a valuable resource for preclinical testing and materials development, providing a first-line assay of biocompatibility that ensures the ability of materials to induce or support vital angiogenesis. Development and application hold considerable promise and benefit for an ageing demographic and regenerative medicine.
